# Genomic and Phenotypic Diversity of Cultivated and Wild Tomatoes with Varying Levels of Heat Tolerance

**DOI:** 10.3390/genes12040503

**Published:** 2021-03-29

**Authors:** Mathieu Anatole Tele Ayenan, Agyemang Danquah, Paterne A. Agre, Peter Hanson, Isaac Kwadwo Asante, Eric Yirenkyi Danquah

**Affiliations:** 1 West Africa Centre for Crop Improvement, College of Basic and Applied Science, University of Ghana, PMB LG30 Legon, Ghana; mayenan@wacci.ug.edu.gh (M.A.T.A.); asanteisaack57@gmail.com (I.K.A.); edanquah@wacci.ug.edu.gh (E.Y.D.); 2 World Vegetable Center, West and Central Africa—Coastal and Humid Regions, IITA-Benin Campus, 08 BP 0932 Cotonou, Benin; 3 Department of Crop Science, Faculty of Agriculture, College of Basic and Applied Sciences, University of Ghana, P. O. Box LG44 Legon, Ghana; 4 International Institute of Tropical Agriculture (IITA), PMB 5320, Ibadan 200001, Nigeria; P.Agre@cgiar.org; 5 Department of Plant Biology and Environmental Science, College of Basic and Applied Sciences, University of Ghana, P. O. Box LG55 Legon, Ghana

**Keywords:** genetic variability, heat tolerance, heritability, SNP markers, tomatoes

## Abstract

Assessment of genetic variability in heat-tolerant tomato germplasm is a pre-requisite to improve yield and fruit quality under heat stress. We assessed the population structure and diversity in a panel of three *Solanum pimpinellifolium* (wild tomatoes) and 42 *S. lycopersicum* (cultivated tomatoes) lines and accessions with varying heat tolerance levels. The DArTseq marker was used for the sequencing and 5270 informative single nucleotide polymorphism (SNP) markers were retained for the genomic analysis. The germplasm was evaluated under two heat stress environments for five yield and flower related traits. The phenotypic evaluation revealed moderate broad-sense heritabilities for fruit weight per plant and high broad-sense heritabilities for fruit weight, number of inflorescences per plant, and number of flowers per inflorescence. The hierarchical clustering based on identity by state dissimilarity matrix and UPGMA grouped the germplasm into three clusters. The cluster analysis based on heat-tolerance traits separated the germplasm collection into five clusters. The correlation between the phenotypic and genomic-based distance matrices was low (*r* = 0.2, *p* < 0.05). The joint phenotypic and genomic-based clustering grouped the germplasm collection into five clusters well defined for their response to heat stress ranging from highly sensitive to highly tolerant groups. The heat-sensitive and heat-tolerant clusters of *S. lycopersicum* lines were differentiated by a specific pattern of minor allele frequency distribution on chromosome 11. The joint phenotypic and genomic analysis revealed important diversity within the germplasm collection. This study provides the basis for efficient selection of parental lines to breed heat-tolerant varieties.

## 1. Introduction

Tomato is one of the major vegetables grown and consumed worldwide [1]. However, tomato production faces several biotic (pests and diseases attack) and abiotic (salinity, heat and drought) constraints [2]. Heat stress is one of the major abiotic stresses limiting tomato production in tropical and sub-tropical regions [3]. Heat stress negatively affects vegetative fresh shoot weight, photosynthetic apparatus, reproductive-related traits including pollen viability, pollen tube growth, and subsequently fruit set, fruit weight, number of fruits, and yield [4,5,6].

Predictions from different climatic scenarios and models agree that the earth’s temperatures are rising [7], which is a major concern for crop production in general, and tomato production in particular [3]. In the tropical and sub-tropical areas where tomato is almost exclusively produced outdoors, by 2100 projected increased temperatures will exacerbate the seasonality of tomato production and decrease favorable tomato production areas [3]. Similarly, a reduction of suitable areas for tomato production was projected to occur worldwide by 2050 [8]. Thus, the importance of improving heat tolerance in crop varieties to adapt to existing and predicted rising temperatures has been receiving increasing attention [9,10,11]. There have been efforts in improving heat tolerance in tomatoes. However, a narrow genetic base has limited breeding for heat tolerance in cultivated tomato [12]. This is partly due to the limited screening of available germplasm for heat tolerance [6]. The success of both conventional and molecular breeding methods depends on genetic variability for the traits of interest. Diversity assessment in cultivated and wild relative crop species provides a foundation for conservation strategies and for breeding program establishment [13]. 

Tomato diversity and performance under heat stress has been extensively assessed using a variety of tools: Phenotypic traits [14,15,16,17,18,19], biochemical markers [18], high throughput molecular markers [17,19,20]. These studies have provided insight into the existing diversity in the cultivated tomatoes and wild relatives and revealed that genetic diversity within each germplasm collection is unique. In addition, none of these studies systematically combined phenotypic and molecular markers in assessing the diversity of their germplasm collection. A joint phenotypic and molecular analysis combines the grouping of germplasm based on traits of importance and the power of single nucleotide polymorphism (SNP) markers (abundant in the genome, independent from growing conditions), which improves the reliability of diversity assessment in a germplasm collection [21,22]. Improving the exploitation of genetic resources for heat tolerance breeding in tomatoes requires a systematic assessment of germplasm collections using a combination of phenotypic and genomic tools [6].

Here, we assembled a germplasm of heat-tolerant and sensitive lines from various genebanks and breeding programs worldwide to initiate a breeding program to improve heat tolerance in tomatoes along with other agronomically important and consumer-preferred traits. This germplasm collection has already been comprehensively characterized using a combination of quantitative and qualitative traits under a semi-controlled environment [23]. However, our knowledge of the genomic diversity in this germplasm is limited. Assessment of genetic diversity in this unique germplasm is a requirement to select parental lines for breeding hybrids adapted to heat stress. Heritability of the heat-tolerance traits and their association under heat stress in tomatoes are not congruent across studies [6]. Knowledge of available diversity, heritability, and association among traits are important to make informed decisions regarding the choice of breeding strategy. The findings of our study will guide other researchers on the choice of parental lines to accelerate genetic gain under heat stress while maintaining a sufficient amount of diversity for sustainable use of the tomato plant genetic resources. This present study aimed to (i) assess the variability and relatedness in the germplasm panel based on genomic and phenotypic traits, and (ii) assess the correlation among the phenotypic traits and their broad-sense heritabilities.

## 2. Materials and Methods

### 2.1. Plant Material

The germplasm collection consisted of 45 lines (improved and unimproved lines, accessions, and wild relatives) (Table 1), from the Tomato Genetics Resource Center (TGRC) of the University of California (Davis), the World Vegetable Center (Taiwan), University of Florida, the Crop Research Institute (Ghana), and commercial and local varieties collected in Ghana and Benin. Three of the 45 lines were *Solanum pimpinellifolium* accessions obtained from the TGRC. The lines were selected based on their response to heat stress in previous studies [6,23].

### 2.2. Phenotypic Evaluation

The germplasm was evaluated under long-term mild heat stress in greenhouse (October 2019 to February 2020 in Legon, Ghana) and in open field (March to June 2020 in Ze, Benin) conditions (Table 2). The experiments were set up in a randomized complete block design with two replications at each location. The number of plants per plot was on average five with a spacing of 60 × 60 cm. Six heat tolerance-related traits were collected to assess the diversity of the germplasm.

Six to nine inflorescences per plot were randomly selected and tagged at the flowering stage. The number of flowers (NFl) and number of fruits (NFr) were counted to estimate the percentage of fruit setting (FS = NFr/NFl) × 100.

The number of fruits and fruit weight per plot were recorded and added up at each fruit picking until termination of the experiment. The average number of fruits per plant (NFP) and fruit weight per plant (FWP) were computed by dividing the total number of fruits and the total fruit weight per plot by the number of plants per plot.

Average fruit weight (FW) was computed by dividing the total fruit weight per plot by the total number of fruits per plot. The association among the traits was assessed using the Pearson method in R 4.0.2 (Foundation for Statistical Computing: Vienna, Austria) [24] and visualized using a correlogram in the corrplot package [25]. The variance components were estimated using a linear model in lmerTest package [26]. 

The linear mixed model is represented as follows in Equation (1):

(1) $$y_{\mathrm{ijk}}=u+{\;G}_{i}+{\;E}_{j}+{\;\mathrm{GE}}_{\mathrm{ij}}+\;R{\left(E\right)}_{\mathrm{jk}}+\;\varepsilon_{\mathrm{ijk}},$$

where 𝐲_𝐢𝐣𝐤_ is the k^th^ observation for the i^th^ genotype in the j^th^ environment, u is the overall mean, $G_{i}$ is the effect of i^th^ genotype, $E_{j}$ is the effect of jth environment, $R{\left(E\right)}_{jk}$ is the effect of the k^th^ replication within the j^th^ environment, $GE_{ij}$ is the effect of the interaction of the i^th^ genotype and j^th^ environment, and $\varepsilon_{ijk}\;$ is the residual effect.

The broad sense heritability (H) was computed on a line mean basis for each trait using the variance components following Equation (2) [27,28]:

(2) $$H^{2}=\frac{\sigma_{g}^{2}}{\sigma_{g}^{2}+\frac{\sigma_{\mathrm{ge}}^{2}}{e}+\frac{\sigma_{e}^{2}}{\mathrm{re}}}\;,$$

where σ^2^_g_ is the genotypic variance, σ^2^_ge_ is the variance of genotype by environment interaction, σ^2^_e_ is the residual or environmental variance, and r and e are the number of replications within the environment and the number of environments, respectively.

Prior to the clustering of the germplasm based on the six traits, we computed the cophenetic coefficient of correlation using a combination of two distance matrices (Euclidean and Mahalanobis) and two clustering algorithms (ward.D2 and UPGMA). The combination of Euclidean distance and UPGMA yielded the highest cophenetic coefficient of correlation (0.918) and was used for the clustering in the package dendextend [29].

### 2.3. Genotyping of the Germplasm Collection

Young leaves were collected into a 96-deep well sample collection plate and sent for genotyping at the Integrated Genotyping Service and Support (IGSS) platform at Biosciences Eastern and Central Africa (BecA-ILRI) Hub in Nairobi, Kenya. DNA was extracted using the Nucleomag Plant Genomic DNA extraction kit. DNA quality and quantity were checked by electrophoresis on 0.8% agarose gel. We implemented the Diversity Arrays Technology protocol for genome complexity reduction, which encompasses digestion of genomic DNA and adapters ligation according to Kilian et al. [30]. The targets were pooled to prepare the library, which was purified using the QIAGEN PCR purification kit. Cluster generation was carried out in cBOT (Illumina, San Diego, CA, USA). The Illumina HiSeq 2500 was used for sequencing. Single-read sequencing runs for 77 bases were performed to sequence libraries. Polymorphism identification and calling, and generation of quality control parameters for selection of polymorphic markers were performed in the secondary pipeline in the KDCompute plug-in platform using DArTSoft14 (Diversity Arrays Technology, Canberra, ACT, Australia). The genomic representation of the sample and the generated SNP markers were aligned to the tomato reference genome SL4.0. The analysis herein was based on the SNP markers.

### 2.4. SNP Filtering Summary

A total of 14,142 DArT SNP markers were generated through DArTSoft14 (Diversity Arrays Technology, Canberra, Australia). After removing SNP which were not mapped to any chromosome, with call rates lower than 80%, missing values higher than 20%, minor allele frequencies lower than 1%, and duplicated physical positions, 5270 SNP markers were retained for the analysis. The lines Fla.7776 (missing value higher than 40%) and LA2854 (heterozygosity higher than 5%) were removed and 43 accessions were used for the genomic analyses.

### 2.5. Genetic Population Structure Analysis

The variant call format (VCF) file generated from DArtSeqTM after quality control was imputed using beagle4 version [31]. For high imputation accuracy, the number of phasing iterations was set as 5 with the Identity by Descent (IBD) set as false and nthreads set as default. The statistics summary including observed and expected heterozygosity, minor allele frequency (MAF), and polymorphic information content (PIC) was estimated using “hardy” and “freq” functions implemented in plink [32]. Mutation transversion and transition were determined using SNiPlay3 [33]. The distribution of the SNPs and their density on the 12 tomato chromosomes were assessed using customized perl script. 

Two multivariate analyses, including admixture for population structure, and cluster analysis with the UPGMA algorithm, were used to assess the genetic diversity. Prior to the UPGMA clustering, the optimal number of clusters was estimated using the elbow method in factoextra package [34]. The dendrogram was visualized in FigTree v1.4.4.

A binary file was generated from plink which was later subjected to Admixture analysis using the adegenet package through the Bayesian information criterion (BIC) [35]. The optimal number of populations was inferred using k-means analysis after varying the number of clusters from 2 to 40. The final admixture was plotted using the barplot function implemented in R 4.0.2 [36]. A line was assigned to a model-based sub-population where more than 80% of its inferred ancestry membership coefficient was derived from the given sub-population [37].

An identity by state (IBS) dissimilarity matrix was generated in TASSEL 5.0 [38]. Prior to the clustering analysis, the best combination of the distance matrix and clustering method was assessed by computing the cophenetic coefficients of correlation in R 4.0.2. The combination of IBS and UPGMA yielded the highest cophenetic coefficient of correlation (CCC = 0.992), compared to IBS and neighbor joining (CCC = 0.987), Euclidean distance and UPGMA (CCC = 0.987), or Euclidean distance and NJ (CCC = 0.947). As a result, the clustering was done using UPGMA. The optimal number of clusters was inferred using the elbow technique in NbClust package [39].

### 2.6. Combined Phenotypic and Genomic Datasets

All the dendrograms were generated using 38 lines for which both phenotypic and genomic data were available. The correlation between phenotypic and genomic dissimilarity matrices was assessed using the Mantel statistic based on the Spearman rank correlation rho method and its significance was computed in the vegan package [40] with 10,000 Monte Carlo permutations. A visual comparison between the phenotypic and genomic-based dendrograms was done using the function tanglegram in package dendextend [29].

The dissimilarity matrices were combined the using analogue package [41]. Hierarchical clustering was done on the combined dissimilarity matrix based on UPGMA using hclust function in R 4.0.2 [36]. The joint dendrogram was generated in the package dendextend [29]. The pairwise fixation index [42] was generated to estimate the genetic differentiation among the clusters inferred from the joint phenotypic and genomic-based matrices using the genet.dist function in the hierfstat package [43]. Except when otherwise specified, all the analyses were done in R 4.0.2 [36]. The clustering analyses (phenotypic, genomic, and combined phenotypic and genomic) were performed without cluster size constraints.

The statistics summary including observed and expected heterozygosity, minor allele frequency (MAF), and polymorphic information content (PIC) for each cluster were estimated using “hardy” and “freq” functions implemented in plink [32]. The estimates of MAF within each cluster were plotted across the genome (physical distances) to visualize genetic variations between the different clusters (highly tolerant, moderately tolerant, moderately susceptible, and highly susceptible) [44].

## 3. Results

### 3.1. Variance Components of the Heat Tolerance Traits and Heritability Estimate

The linear mixed model revealed that the residual and genotype by environment interaction variances were much higher than the genotypic variance for fruit weight per plant contrary to that of the other traits (Table 3). Fruit weight per plant recorded the lowest broad sense heritability (H^2^ = 0.41), whereas the number of flowers per inflorescences had the highest broad sense heritability value (H^2^ = 0.90). The fruit set percentage and number of fruits per plant had moderate broad sense heritability.

### 3.2. Correlation Analysis Among the Quantitative Traits

**Figure 1:**
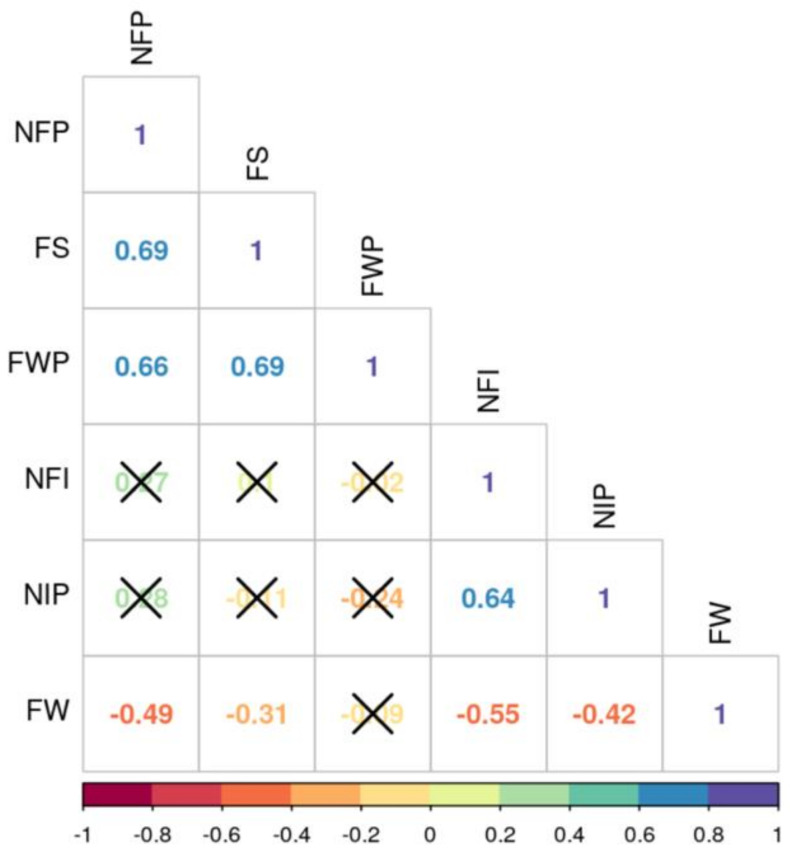
Correlations among the traits in a tomato germplasm collection assessed under heat stress. NFP: Number of fruits per plant, FS: Fruit setting percentage, FWP: Fruit weight per plant, NFI: Number of flowers per inflorescence, NIP: Number of inflorescences per plant, FW: Fruit weight. The figure shows the Pearson correlation coefficients between selected traits. The color of the number reflects the strength of the correlation. The non-significant correlations, with a *p*-value above 0.05, are indicated with a cross in the individual cells.

Fruit weight per plant (FWP) was strongly and positively associated with number of fruits per plant (NFP, r = 0.66) and fruit setting percentage (FS, r = 0.69) (Figure 1). NFP was positively correlated with FS. Flower parameters (number of inflorescences per plant and number of flowers per inflorescence) were positively correlated but both showed non-significant associations with fruit yield components including average fruit weight (FW), NFP, and FWP. FW was negatively associated with NFP.

### 3.3. Phenotypic Based-Clustering Analysis of the Tomato Lines

**Figure 2:**
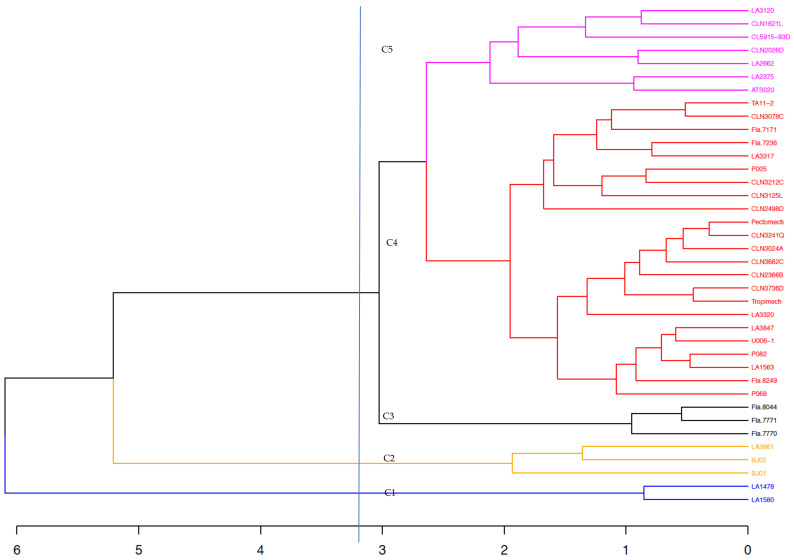
Clustering of the tomato collection based on Euclidean distance and UPGMA using phenotypic traits. C1, C2, C3, C4, and C4 denotes clusters 1, 2, 3, 4, and 5, respectively. Each color represents a cluster.

The phenotypic based-clustering analysis grouped the germplasm collection into five clusters of unequal size (Figure 2). Cluster 1 is composed exclusively of the two *S. pimpinellifolium* accessions with high sensitivity to heat stress. Cluster 2 is composed of three *S. lycopersicum* lines that showed high levels of tolerance to heat stress (Table S1). Cluster 3 grouped three of the large fruited (average fruit weight = 114.71 ± 10.36 g) improved lines from the University of Florida with moderate sensitivity to heat stress. Cluster 4 had the highest membership (23 lines), with lines from diverse origins (WorldVeg, University of Florida, Ghana, TGRC). Accessions in this cluster showed low tolerance to heat stress with an average fruit set percentage and fruit weight per plant of 13.82 ± 7.79% and 347.09 ± 225.52 g/plant, respectively. Cluster 5 grouped moderately tolerant lines with an average fruit set percentage and fruit weight per plant of 32.88 ± 9.89% and 564.58 ± 220.69 g/plant, respectively (Table S1).

### 3.4. Characteristics of the 5270 SNPs in the Germplasm Collection

**Figure 3:**
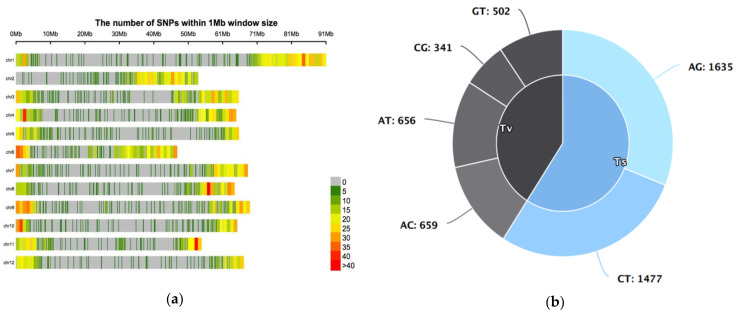
(**a**) Genome-wide single-nucleotide polymorphism (SNP) distributions and density of 5270 SNPs detected by DArTseq on the 12 chromosomes; (**b**) types of 5270 SNPs mutations detected by DArTseq of 43 *S. lycopersicum* and *S. pimpinellifolium* inbred lines and accessions on the 12 chromosomes.

The 5270 SNPs were distributed across the 12 tomato chromosomes (Figure 3). The number of SNPs per chromosome varied from 318 (chromosomes 10 and 12) to 640 (chromosome 1) with an average of 439 SNPs per chromosome. The SNP types analysis revealed that transition mutations (CT and AG) were higher (3112; 59%) than transversion mutations (2158; 41%) (Figure 3). AG transition was slightly higher than CT transition. Regarding the transversion mutations, AT and AC occurred almost at the same rate but higher than the occurrence rate of CG and GT. The MAF ranged from 0.01 to 0.49 with an average of 0.073 (Table S2), while 0.107 was obtained for PIC (Table S2). The average observed heterozygosity of the SNPs was 0.011, which was far lower than the average expected heterozygosity of 0.125 (Table S2).

### 3.5. Population Structure

**Figure 4:**
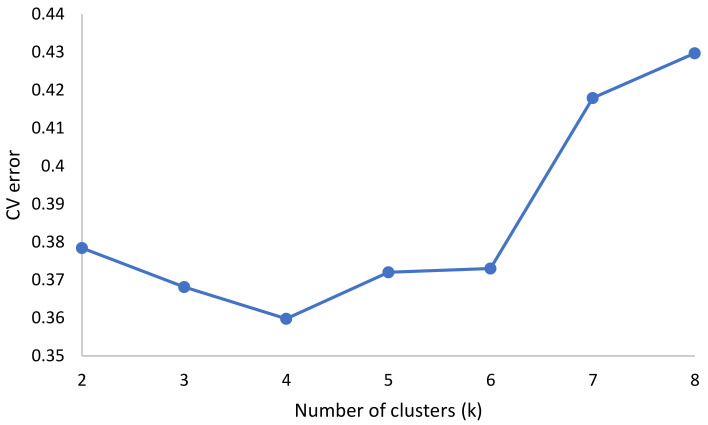
Variation of cross-validation (CV) error as a function of number of clusters.

**Figure 5:**
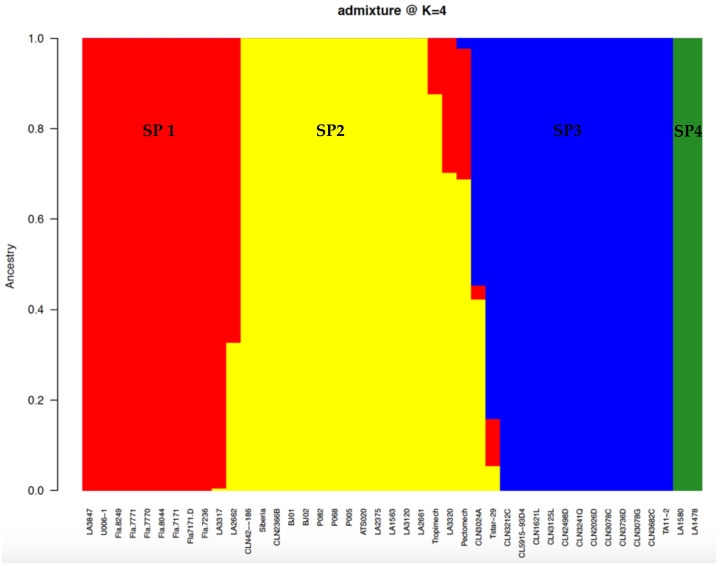
Population structure in a tomato germplasm collection. Each entry’s genome is divided into colored segments following the proportion of the estimated ancestry membership coefficient in the four subpopulations. Sub-populations SP1, SP2, SP3, and SP4 are represented by red, yellow, magenta, and green, respectively.

The cross-validation (CV) error outputs from Admixture showed an elbow from k = 2 to k = 4, suggesting that there were four sub-populations in the germplasm collection (Figure 4). Based on the cutoff point (0.80) of the ancestry membership coefficient, 39 lines were assigned to one of the four sub-populations (Figure 5). Sub-population 2 had the highest membership (36%) followed by sub-population 3 (33%), sub-population 1 (26%) and sub-population 4 (5%) (Figure 5). All the improved lines (Fla.) from the University of Florida were clustered in sub-population 1, with four lines from the TGRC. Sub-population 2 was composed of lines collected from Benin and Ghana, including the commercial variety Tropimech. Some improved lines from the World Vegetable Center were also found in this sub-population. Sub-population 3 consisted exclusively of improved lines from the World Vegetable Center. Sub-population 4 was composed exclusively of the two S. pimpinellifolium accessions LA1580 and LA1478 and showed no admixture with the S. lycopersicum lines. Four admixed lines were detected between the S. lycopersicum sub-populations. CLN024A and Pectomech were admixtures between sub-populations 1, 2, and 3, and LA3320, and LA2662 were admixtures between sub-populations 1 and 2.

### 3.6. Genetic Distances Among the Tomato Lines Based on the 5270 SNPs and Hierarchical Clustering

The genetic distance was computed as 1 - IBS, with IBS defined as the probability that alleles drawn at random from two individuals at the same locus are the same. To identify duplicates in the germplasm, we intentionally included duplicated samples (Fla7171 and Fla7171.D). We assumed that duplicate accessions in the germplasm collection would have distances equal to or lower than the genetic distance between Fla7171 and Fla7171.D, i.e., 0.008. The pairwise distance between the accessions revealed that all the accessions in the germplasm collection were unique since there was no pair with distance equal to or lower than 0.008 (Table S3). The highest genetic distance values were observed between the *S. pimpinellifolium* accessions and the *S. lycopersicum* accessions. LA1478 (*S. pimpinellifolium*) and CLN3078C had the highest genetic distance (0.556). The distance between the two *S. pimpinellifolium* accessions (LA1580 and LA1478) was 0.232. Among the cultivated tomato, LA1563 and CLN3682C had the highest genetic distance (0.189) while LA3847 (NCHS-1) and U006-1, an off-type isolated from LA1563 (obtained from TGRC), had the lowest genetic distance (0.01) (Table S3). 

**Figure 6:**
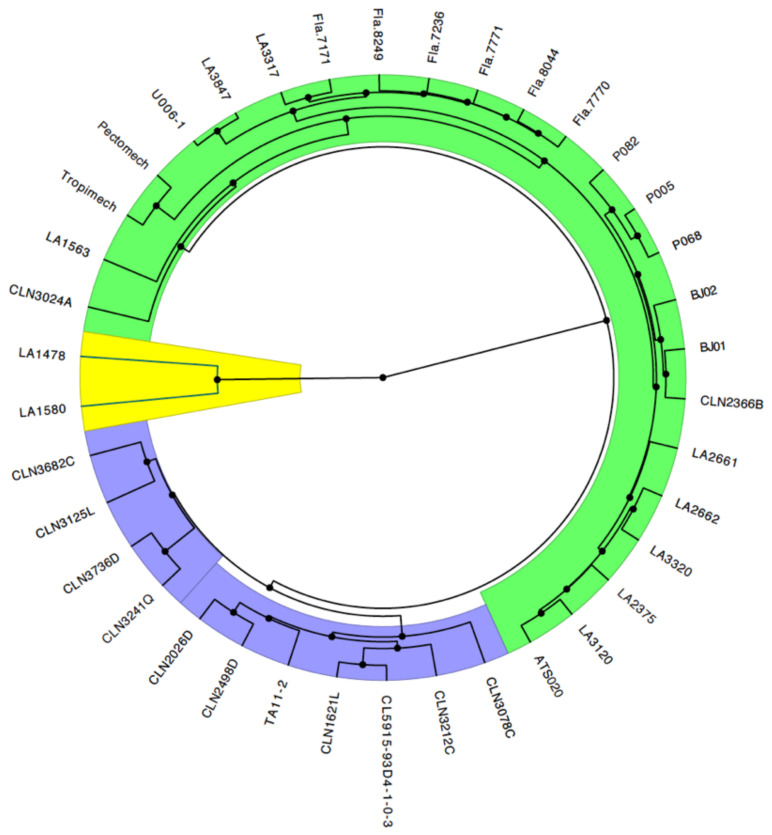
Clustering of the germplasm based on identity by state (IBS) dissimilarity matrix and UPGMA of a tomato germplasm collection. Cluster 1, Cluster 2, and Cluster 3 are highlighted in yellow, purple, and green, respectively.

UPGMA clustered the lines/accessions into three groups (Figure 6). The two *S. pimpinellifolium* accessions formed a cluster (Cluster 1), separated from the *S. lycopersicum* lines. The *S. lycopersicum* lines were grouped into two clusters, of which one (Cluster 2) was exclusively composed of improved lines from the World Vegetable Center (Figure 6). Cluster 3 had the largest number of lines from various origins including the TGRC, University of Florida, Ghana, Benin, and the World Vegetable Center. Within this cluster, there were sub-groups, which were consistent with the pedigree information. For instance, the “Fla.” lines from the University of Florida were clustered together similar to the commercial varieties (Pectomech and Tropimech), and the lines (P005, P068, P082) from the Crops Research Institute, Ghana were clustered together. Lines from the World Vegetable Center had a relatively broad genetic base as they were distributed across two of the three clusters, conversely to lines from the University of Florida and Crop Research Institute, Ghana, which were only found in one cluster.

### 3.7. Combined Phenotypic and Genomic Clustering

The Mantel test revealed a low and significant correlation (*r* = 0.2, *p* < 0.05) between the genomic- and phenotypic-based distance matrices (Table S4). However, there were three common sub-trees, namely LA1478 and LA1580, LA3847 and U006-1, and Fla.7770, Fla.8044, and Fla.7771, between the phenotypic and genomic-based dendrograms (Figure S1). Due to the low correlation between the two dendrograms, the phenotypic- and genomic-based distance matrices were combined for a joint clustering analysis. The clusters formed based on the phenotypic and those from the genomic data were positively correlated with the clusters formed based on the combination of phenotypic and genotypic data. However, the correlation between the phenotypic-based distance matrix and the combined phenotypic and genotypic distance matrix (*r* = 0.91, *p* < 0.001) was higher than that of the genomic-based distance matrix and the combined phenotypic and genomic distance matrix (*r* = 0.54, *p* < 0.001) (Table S4).

**Figure 7:**
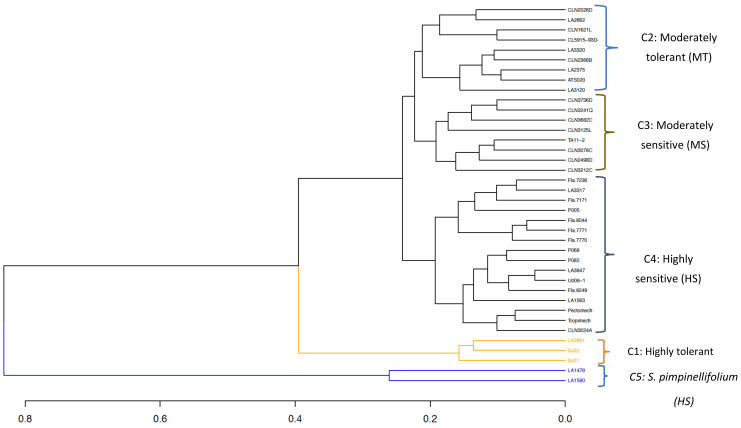
Clustering based on combined phenotypic and genomic distance matrices of a tomato germplasm collection. C denotes Cluster.

The joint clustering analysis grouped the germplasm collection into five clusters, ranging from highly sensitive to highly tolerant groups (Figure 7). Cluster 1 was composed of three highly heat-tolerant lines with the highest fruit set percentage (47.52 ± 5.21%), fruit weight per plant, and number of fruits per plant (Table 3). Among the *S. lycopersicum*, lines in Cluster 1 had the highest and the lowest number of inflorescences per plant and individual fruit weight, respectively. Cluster 2 (moderately tolerant) grouped nine lines from the World Vegetable Center, Ghana and TGRC and was characterized by a moderate fruit set percentage (27.60 ± 13.37%), fruit weight per plant and number of fruits per plant. Cluster 2 also had the highest number of flowers per inflorescence (Table 3). The highly sensitive group (cluster 4, 16 lines) was composed of the Fla. lines from the University of Florida, the three lines from Crop Research Institute, Ghana, the two commercial varieties, and some accessions from the TGRC (Figure 7). This group had the highest fruit weight (61.00 ± 30.56 g) but showed low performance for all the other traits (Table 4).

Except for observed heterozygosity, Cluster 5 had the highest values for the genomic parameters (MAF, PIC, and expected heterozygosity) followed by Clusters 3, 2, 4, and 1, in this order (Table 4).

The plots of the MAF for the different clusters showed a different pattern of distribution of MAF over the whole genome (Supplementary File S1). We observed specific patterns on chromosomes 6, 9, and 11. Subsequently, the MAF distribution patterns of each cluster on chromosomes 6, 9, and 11 were plotted (File S1). Cluster 3 showed a unique pattern on chromosome 6 relative to the other clusters. Cluster 2 showed unique pattern on chromosome 9. MAF distribution pattern on chromosome 11 distinguished heat-tolerant lines (Clusters 1 and 2) from the heat-sensitive lines (Clusters 3 and 4).

### 3.8. Differentiation Among Populations

We computed the pairwise fixation index among the four sub-populations. The fixation index measured the decrease in heterozygosity due to subdivision within a population. The high differentiation index between Cluster 5 was composed of the *S. pimpinellifolium* lines and the four clusters formed by the *S. lycopersicum* lines. Clusters 4 and 5 and Clusters 1 and 2 had the highest (0.83) and the lowest (0.10) differentiation indices, respectively (Table 5). Among the *S. lycopersicum* lines, Cluster 3, composed of eight of the WorldVeg lines, had the highest differentiation indices with the other clusters.

## 4. Discussion

### 4.1. SNP Quality

In this study, we assessed the diversity in a germplasm collection assembled to improve heat tolerance in tomato using a combination of SNP markers and heat tolerance-related traits.

The DArTseq genotyping revealed 5270 informative SNPs, which were unequally distributed among and within chromosomes. Telomeric regions had higher SNP density compared to peri-centromeric regions. Distal euchromatin regions (telomeric regions) were more densely covered with genes and had higher recombination rates compared to peri-centromeric heterochromatin regions [45] results that were similar to findings in pearl millet [46].

### 4.2. Genetic Diversity in the Germplasm Collection

We assessed the level of genetic diversity in the germplasm collection through polymorphic information content (PIC), minor allele frequency (MAF), and the expected (He) and observed heterozygosity (Ho). The average PIC (0.107) and expected heterozygosity values (0.125) were in the range of those reported by Sim et al. [44] in different classes of tomatoes using SNP markers.

The expected heterozygosity for the SNPs was higher than the observed heterozygosity, confirming the high autogamous nature of cultivated tomato and including the two *S. pimpinellifolium* accessions [47]. This high homozygosity implies that no further selfing of the lines in the germplasm collection is needed before using them in genetic studies, for hybrid or population development. 

The genetic distance between the *S. pimpinellifolium* accessions and the *S. lycopersicum* lines was higher than that of the intra-specific genetic distances, reinforcing the notion of genetic divergence between the two species. This is consistent with the high differentiation indices observed between the sub-population composed of the *S. pimpinellifolium* lines and those of the *S. lycopersicum* lines. The genetic differentiation between the different clusters could be due to the founder effect or human selection for diverse growing environments, production systems, and market preferences [44]. Clear differentiation based on morphological markers and microsatellite markers between *S. lycopersicum* and *S. pimpinellifolium* was previously reported [48]. Interestingly, *S. pimpinellifolium* is the closest wild relative of the cultivated tomato and interspecific crossing is easy and resulting in fertile offspring [48,49]. This offers an opportunity to transfer favorable alleles for heat-tolerance traits like high pollen viability [16,23], or a high number of fruits per plant from *S. pimpinellifolium* to *S. lycopersicum*. However, harnessing traits of interest from wild relative species may be limited by linkage drag [50]. In this regard, development and evaluation of inbred backcross lines can help identify and transfer valuable quantitative trait loci (QTL) from non-adapted to elite lines, and increase the power to detect QTL with minor effects [50,51,52].

No duplicate lines were found in this collection and all were distinct. Considering the diversity of heat-tolerant lines in the germplasm collection and their origins, assembling representative lines from each of the clusters could form a mini-core collection for heat tolerance breeding and research.

The grouping of the germplasm collection based on population structure analysis was not consistent with their heat tolerance status. Sub-population 2 grouped both heat-tolerant (e.g., LA2661, BJ01, ATS020) and heat-sensitive accessions (e.g., P005, P068, Tropimech) [23]. This finding was supported by the inconsistency between genomic-based and phenotypic-based clustering. This is not surprising since SNP markers are neutral, suggesting that the lines were not discriminated based on their response to given stress or specific agronomic traits. Similar inconsistencies were reported in many other crops such as water yam [21], chickpea [22], and sweet sorghum [53]. This highlights the importance of combining phenotypic and molecular information while selecting parental lines in breeding programs to take into account their phenotypic performance and their genetic background. Phenotypic traits have the advantage of revealing the performance of lines but have limited polymorphism and they are subjected to changes in environmental conditions. The phenotypic markers are adaptive traits and subjected to natural and artificial selection conversely to molecular markers [54]. On the other hand, SNP markers are highly diverse and polymorphic but neutral. Harnessing the advantages of phenotypic and molecular markers improves the grouping of lines in a germplasm collection [53], which provides valuable information for the selection of parental lines to realize and sustain genetic gain. Furthermore, clustering inbred lines is a first step in developing heterotic groups in a large germplasm collection where testers are not readily available. Three to four representative lines could be selected from each cluster formed by the *S. lycopersicum* lines and intercrossed. Subsequently, the hybrids should be evaluated in several environments along with their parents to identify promising crosses and group the lines to form heterotic groups based on specific and general combining abilities [55]. 

Consistent with our previous evaluation [23], five clusters were formed based on the phenotypic traits. The lines showed variable responses to heat stress ranging from highly sensitive to highly tolerant. The genetic parameters assessed were higher in the *S. pimpinellifolium* cluster. This is consistent with the previous finding by Sim et al. [44] who reported higher expected heterozygosity and PIC in the *S. pimpinellifolium* sub-population in comparison with cultivated tomato. Higher genetic diversity in *S. pimpinellifolium* compared to *S. lycopersicum* was expected because the former can exhibit facultative outcrossing, with wide geographical distribution in their native region [56]. The specific distribution patterns observed for MAF on chromosomes 6, 9, and 11 suggest that these chromosomes could be under diversifying selection relative to other regions of the genome [44]. The MAF distribution on chromosome 11 distinguishes heat-tolerant clusters from heat-sensitive ones. Interestingly, meta-quantitative traits’ loci were detected on chromosome 11 for style length, style protrusion, and pollen viability, which are key heat-tolerance traits [6,57].

The joint genomic and phenotypic diversity assessment revealed relatively high genetic variability in the germplasm collection, challenging the assumption of a narrow genetic base of heat tolerance in tomato. The clustering based on the combination of phenotypic and genomic dataset grouped the germplasm into well-defined clusters regarding their origins and their reaction to heat stress. LA2661 (Nagcarlang), a well-known heat-tolerant was grouped with two accessions from Benin, BJ01 and BJ02, which were previously reported as heat-tolerant [23]. These three lines are large indeterminates producing lots of inflorescences and setting many small fruits (less than 15 g). LA2662, LA3317, CLN1621L, CL5915-93D-1-0-3, LA2662, and ATS020 in the group of moderate heat-tolerant are known for their heat tolerance status [6,12,15]. The Fla. lines were previously reported as heat-tolerant [58,59]; however, under both greenhouse [21] and field evaluation they were sensitive to heat stress. This suggests that reaction to heat is sometimes environment-specific, especially in regard to the intensity and duration of the heat [60] and other climatic factors such as relative humidity [61]. The Fla. lines which have large fruit size under the heat stress conditions could be more heat tolerant than other large-fruited lines. In fact, a recent finding revealed that tomato traits associated with heat tolerance depend on the fruit size [62]. 

The grouping of the germplasm collection into different clusters is a basis for the development of multi-parental populations. Actually, development of multi-parental populations by crossing lines from the different heat-tolerant groups will help decipher the genetic basis of heat tolerance in tomato and provide better understanding of the molecular basis of fruit weight under heat stress.

### 4.3. Broad Sense Heritability and Trait Associations

Moderate to high broad-sense heritabilities found for number of inflorescences per plant, number of flowers per inflorescence, number of fruits per plant, fruit weight, and fruit setting percentage implied that the lines had a consistent response to heat stress across the environments (greenhouse and outdoor). Broad-sense heritability for the number of flowers per inflorescence was consistent with previously reported values (0.712–0.86) [63]. Similarly, the high broad-sense heritability recorded for fruit weight was consistent with the reported value under permissive temperature conditions (0.97) [64]. The fruit setting percentage, number of fruits per plant, and fruit weight per plant had low to moderate broad-sense heritability, which was due to higher residual and genotype by environment interaction variances compared to the genotypic variance. As a result, reliable selection can be done for fruit weight and flowers related traits (number of inflorescences per plant and number of flowers per inflorescence) under few testing environments.

The strong and positive associations between fruit weight per plant, fruit setting percentage, and the number of fruits per plant, and the negative association between fruit weight and the number of fruits per plant were consistent with previous findings [6]. This suggests that the positive correlation among weight per plant, fruit setting percentage, and the number of fruits per plant and the negative association between fruit weight and the number of fruits per plant is underlined by genetic factors including the effects of linkage disequilibrium between loci or pleiotropy.

## 5. Conclusions

This study reported significant genetic variability for heat tolerance traits in the tomato germplasm collection. We observed moderate to high broad-sense heritabilities for the heat-tolerance traits that will guide decision-making upon the number of locations required for efficient and effective phenotyping and selection. Strong and significant associations exist between heat-tolerance traits that could be exploited for indirect selection. We identified varying levels of heat-tolerant lines from each of the clusters that are potential parental lines for genetic analysis and line development. The clustering of the germplasm collection offers an opportunity to develop heterotic groups for hybrid breeding to improve heat tolerance in tomato.

## Figures and Tables

**Table 1:** Origins of accessions/lines evaluated.

Lines/Accessions	Species	Heat Tolerance Status	Sources of Accessions/Lines
Fla.7770	*S. lycopersicum*	Sensitive	University of Florida
Fla.7771	*S. lycopersicum*	Sensitive	University of Florida
Fla.7171	*S. lycopersicum*	Sensitive	University of Florida
Fla.8044	*S. lycopersicum*	Sensitive	University of Florida
Fla.7236	*S. lycopersicum*	Sensitive	University of Florida
Fla.8249	*S. lycopersicum*	Sensitive	University of Florida
Fla.7776	*S. lycopersicum*	Sensitive	University of Florida
LA2661 (Nagcarlang)	*S. lycopersicum*	Tolerant	TGRC, UC Davis
LA3317 (Campbell 28)	*S. lycopersicum*	Tolerant	TGRC, UC Davis
LA2662 (Saladette)	*S. lycopersicum*	Tolerant	TGRC, UC Davis
LA3120 (Malintka 101)	*S. lycopersicum*	Tolerant	TGRC, UC Davis
LA1563	*S. lycopersicum*	Sensitive	TGRC, UC Davis
LA2375 (San Marzano)	*S. lycopersicum*	Tolerant	TGRC, UC Davis
LA3320 (Hotset)	*S. lycopersicum*	Sensitive	TGRC, UC Davis
LA0657 (Beaverlodge)	*S. lycopersicum*	Tolerant	TGRC, UC Davis
LA3847 (NCHS-1)	*S. lycopersicum*	Sensitive	TGRC, UC Davis
LA1580	*S. pimpinellifolium*	Sensitive	TGRC, UC Davis
LA2854	*S. pimpinellifolium*	Sensitive	TGRC, UC Davis
LA1478	*S. pimpinellifolium*	Sensitive	TGRC, UC Davis
U006-1	*S. lycopersicum*	Sensitive	Off-type isolated from LA1563 during seed multiplication
CLN3212C	*S. lycopersicum*	Tolerant	WorldVeg
CLN1621L	*S. lycopersicum*	Tolerant	WorldVeg
CLN3125L	*S. lycopersicum*	Tolerant	WorldVeg
CLN3241Q	*S. lycopersicum*	Tolerant	WorldVeg
CLN3078C	*S. lycopersicum*	Tolerant	WorldVeg
CLN3682C	*S. lycopersicum*	Tolerant	WorldVeg
CLN3024A	*S. lycopersicum*	Tolerant	WorldVeg
CL5915-93D4-1-0-3	*S. lycopersicum*	Tolerant	WorldVeg
CLN2366B	*S. lycopersicum*	Tolerant	WorldVeg
CLN2498D	*S. lycopersicum*	Tolerant	WorldVeg
CLN2026D	*S. lycopersicum*	Tolerant	WorldVeg
CLN3736D	*S. lycopersicum*	Sensitive	WorldVeg
TA11-2	*S. lycopersicum*	Tolerant	Off-type isolated from CLN2498D during seed multiplication
BJ01	*S. lycopersicum*	Tolerant	Benin
BJ02	*S. lycopersicum*	Tolerant	Benin
ATS020	*S. lycopersicum*	Tolerant	Ghana
P082	*S. lycopersicum*	Sensitive	Ghana
P005	*S. lycopersicum*	Sensitive	Ghana
P068	*S. lycopersicum*	Sensitive	Ghana
WACI1	*S. lycopersicum*	Tolerant	Ghana
Tropimech	*S. lycopersicum*	Sensitive	Commercial OPV variety
Pectomech	*S. lycopersicum*	Sensitive	Commercial OPV variety
Siberia	*S. lycopersicum*	Unknown*	WorldVeg
Tstar-29-4-3-12-6-11-4	*S. lycopersicum*	Unknown*	WorldVeg
CLN4220F5-186	*S. lycopersicum*	Unknown*	WorldVeg

* The reaction to heat stress of this line is unknown under the target environments.

**Table 2:** Ranges of temperatures and relative humidities during the tomato evaluation.

Locations	Range of Night Temperatures (°C)	Range of Daytime Temperatures (°C)	Range of Night Relative Humidity (%)	Range of Day Relative Humidity (%)
Legon (greenhouse)	24.83–27.78	27.19–33.30	92.80–100.00	65.95–89.86
Ze (open field)	25.3–32.00	27.60–33.70	53.50–95.50	42.00–82.00

**Table 3:** Estimated variance components for the traits across locations.

Table	NIP	NFI	NFP	FW	FWP	FS
$\sigma_{g}^{2}$	41,176	3.39	684.48	716.10	50,747	118.23
$\sigma_{ge}^{2}$	12,890	0.37	616.71	147.30	113,896	101.73
$\sigma_{e}^{2}$	6,607	0.81	216.67	207.10	61,459	64.20
Broad sense heritability	0.84	0.90	0.65	0.85	0.41	0.64

NFP: Number of fruits per plant, FS: Fruit setting percentage, FWP: Fruit weight per plant, NFI: Number of flowers per inflorescence, NIP: Number of inflorescences per plant, FW: Fruit weight. $\sigma_{g}^{2}$: Genotypic variance; $\sigma_{ge}^{2}$: Genotype by environment interaction variance; $\sigma_{e\;}^{2}$: Environmental variance.

**Table 4:** Characteristics of the clusters formed based on combined genomic and phenotypic datasets.

	Traits	Cluster 1 (Highly Tolerant)	Cluster 2 (Moderately Tolerant)	Cluster 3 (Moderately Sensitive)	Cluster 4 (Highly Sensitive)	Cluster 5 (*S. pimpinellifolium*, Highly Sensitive)
	Number of flowers per inflorescence	6.00 ± 0.00	7.00 ± 1.00	5.00 ± 1.00	4.00 ± 1.00	11.00 ± 0.00
	Fruit set percentage (%)	47.52 ± 5.21	27.60 ± 13.37	20.17 ± 5.32	11.15 ± 6.39	1.78 ± 0.70
Phenotypic parameters	Individual fruit weight (g)	11.60 ± 1.82	27.13 ± 7.50	36.08 ± 12.84	61.00 ± 30.56	0.69 ± 0.36
	Fruit weight per plant (g)	1347.05 ± 275.92	537.11 ± 204.58	432.99 ± 224.39	300.13 ± 206.13	9.21 ± 0.14
	Number of inflorescences per plant	121.00 ± 47.00	39.00 ± 19.00	36.00 ± 7.00	32.00 ± 8.00	921 ± 62
	Number of fruits per plant	114.00 ± 15.00	23.00 ± 15.00	14.00 ± 4.00	6.00 ± 4.00	15.00 ± 7.00
	O.HET	0.01	0.01	0.01	0.01	0.04
Genotypic parameters	E.HET	0.04	0.06	0.08	0.06	0.14
	MAF	0.03	0.04	0.06	0.04	0.13
	PIC	0.03	0.05	0.07	0.05	0.11

O.HET: Observed heterozygosity, E.HET: Expected heterozygosity, MAF: Minor allele frequency, PIC: Polymorphism information.

**Table 5:** Pairwise Fst between populations suggested by Admixture.

	Cluster 1	Cluster 2	Cluster 3	Cluster 4
Cluster 2	0.10			
Cluster 3	0.41	0.26		
Cluster 4	0.20	0.14	0.41	
Cluster 5	0.74	0.79	0.75	0.83

## Data Availability

The data presented in this study are available on request from the corresponding authors. The data are not publicly available due to the confidentiality agreement on some of the germplasm.

## Supplementary Materials

The following are available online at https://www.mdpi.com/article/10.3390/genes12040503/s1, Table S1: Phenotypic characteristics of clusters formed based on both phenotypic traits and combined SNP and phenotypic traits in *S. lycopersicum* and *S. pimpinellifolium* germplasm collection; Table S2: Expected heterozygosity, observed heterozygosity, polymorphism information content (PIC), and minor allele frequency (MAF) of 5270 SNPs detected by DArTseq of 43 *S. lycopersicum* and *S. pimpinellifolium* lines on the 12 chromosomes; Table S3: Genetic distance (1-IBS) among 43 *S. lycopersicum* and *S. pimpinellifolium* lines based on 5270 SNPs detected by DArTseq; Table S4: Strength and significance of correlation between phenotypic-based and combined phenotypic- and genomic-based distance matrices of a tomato germplasm collection; Figure S1: Tanglegram comparing membership of clusters based on SNP markers with that based on combined SNP markers and phenotypic traits of *S. lycopersicum* and *S. pimpinellifolium* germplasm collection; File S1: Distribution patterns of minor allele frequency across the clusters formed based on combined SNP markers and phenotypic traits.
