# Supplementary material for: Genomic and Phenotypic Diversity of Cultivated and Wild Tomatoes with Varying Levels of Heat Tolerance
Source: Genes (Basel). 2021 Mar 29;12(4):503. doi: 10.3390/genes12040503 (PMC8067180; doi:10.3390/genes12040503)
Supplement: Supplementary file 1 [file genes-12-00503-s001.zip › Fig S1.docx]

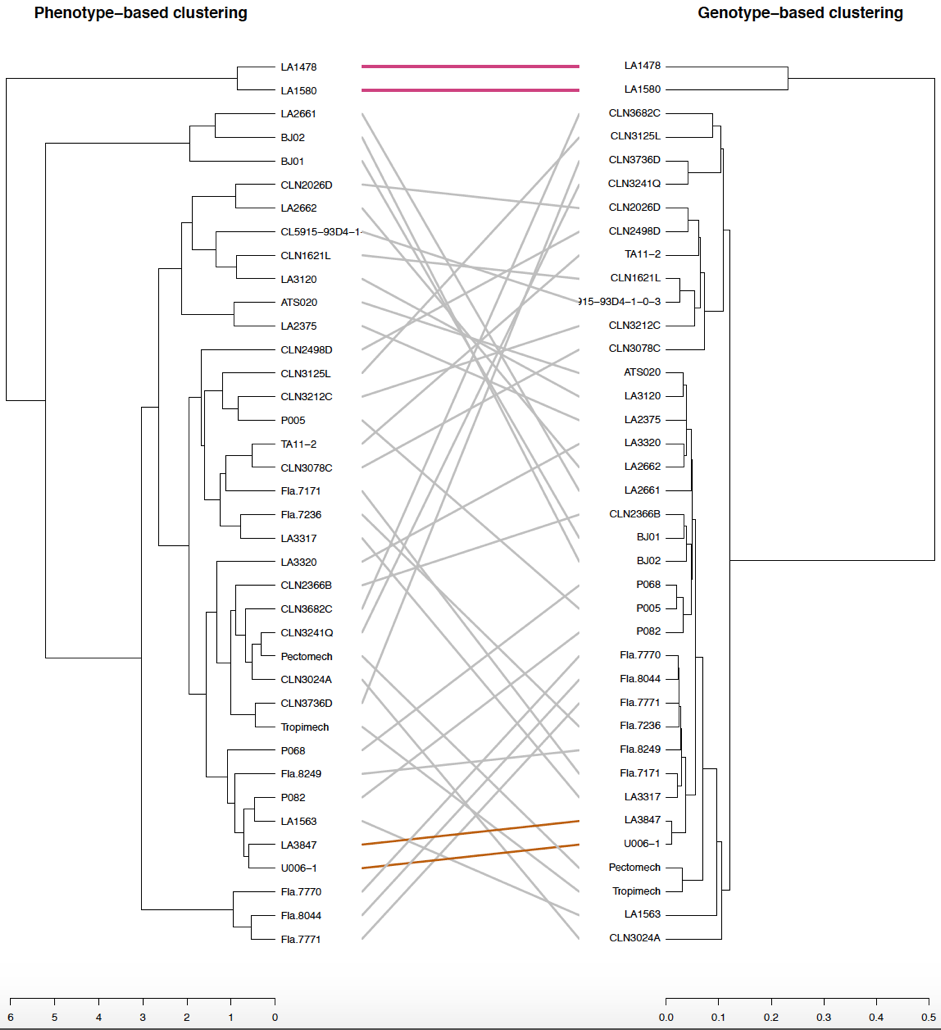


**Figure 1.** Comparison between phenotypic-based and genomic-based dendrogram of tomato germplasm collection.
