# Supplementary material for: Genomic and Phenotypic Diversity of Cultivated and Wild Tomatoes with Varying Levels of Heat Tolerance
Source: Genes (Basel). 2021 Mar 29;12(4):503. doi: 10.3390/genes12040503 (PMC8067180; doi:10.3390/genes12040503)
Supplement: Supplementary file 1 [file genes-12-00503-s001.zip › Table S4.docx]

**Table S4:** Strength and significance of correlation between the distance matrices (DM)

|  | **Phenotypic and genotypic DM** | **Phenotypic and combined DM** | **Genotypic and combined DM** |
| --- | --- | --- | --- |
| **Mantel statistic r** | 0.2 | 0.9093 | 0.5402 |
| **Significance** | 0.047 | 9.999e-05 | 9.999e-05 |

combined=combination of phenotypic and genotypic distance matrices
